# Human Stem Cell‐Derived Endothelial‐Hepatic Platform for Efficacy Testing of Vascular‐Protective Metabolites from Nutraceuticals

**DOI:** 10.5966/sctm.2016-0129

**Published:** 2016-10-07

**Authors:** Balakrishnan Chakrapani Narmada, Yeek Teck Goh, Huan Li, Sanjay Sinha, Hanry Yu, Christine Cheung

**Affiliations:** ^1^ Institute of Molecular and Cell Biology, Proteos, Singapore; ^2^ Institute of Bioengineering and Nanotechnology, Nanos, Singapore; ^3^ The Anne McLaren Laboratory of Regenerative Medicine, Wellcome Trust‐Medical Research Council Cambridge Stem Cell Institute, University of Cambridge, Cambridge, United Kingdom; ^4^ Division of Cardiovascular Medicine, Addenbrooke's Hospital, University of Cambridge, Cambridge, United Kingdom; ^5^ Department of Physiology, Yong Loo Lin School of Medicine, National University of Singapore, Singapore; ^6^ Mechanobiology Institute, Singapore; ^7^ Singapore‐MIT Alliance for Research and Technology, BioSyM, Singapore; ^8^ Lee Kong Chian School of Medicine, Nanyang Technological University, Singapore

**Keywords:** Human pluripotent stem cells, Endothelial cells, Hepatocytes, Coculture, Metabolism, Nutraceutical testing

## Abstract

Atherosclerosis underlies many cardiovascular and cerebrovascular diseases. Nutraceuticals are emerging as a therapeutic moiety for restoring vascular health. Unlike small‐molecule drugs, the complexity of ingredients in nutraceuticals often confounds evaluation of their efficacy in preclinical evaluation. It is recognized that the liver is a vital organ in processing complex compounds into bioactive metabolites. In this work, we developed a coculture system of human pluripotent stem cell‐derived endothelial cells (hPSC‐ECs) and human pluripotent stem cell‐derived hepatocytes (hPSC‐HEPs) for predicting vascular‐protective effects of nutraceuticals. To validate our model, two compounds (quercetin and genistein), known to have anti‐inflammatory effects on vasculatures, were selected. We found that both quercetin and genistein were ineffective at suppressing inflammatory activation by interleukin‐1β owing to limited metabolic activity of hPSC‐ECs. Conversely, hPSC‐HEPs demonstrated metabolic capacity to break down both nutraceuticals into primary and secondary metabolites. When hPSC‐HEPs were cocultured with hPSC‐ECs to permit paracrine interactions, the continuous turnover of metabolites mitigated interleukin‐1β stimulation on hPSC‐ECs. We observed significant reductions in inflammatory gene expressions, nuclear translocation of nuclear factor κB, and interleukin‐8 production. Thus, integration of hPSC‐HEPs could accurately reproduce systemic effects involved in drug metabolism in vivo to unravel beneficial constituents in nutraceuticals. This physiologically relevant endothelial‐hepatic platform would be a great resource in predicting the efficacy of complex nutraceuticals and mechanistic interrogation of vascular‐targeting candidate compounds. Stem Cells Translational Medicine *2017;6:851–863*

**Significance Statement**

Blood vessel diseases underlie a spectrum of debilitating conditions. Nutrition and biologics are increasingly being explored as a strategy to restore vascular health. Unlike the traditional small‐molecule drugs, the complexity of constituents in nutraceuticals presents challenges in predicting their potency in animal testing. This article describes the first human stem cell‐based vascular‐liver coculture system that allows metabolism of nutraceuticals into their vascular‐protective ingredients. This work is significant on several fronts. The liver component enables processing of complex nutraceuticals into metabolites, which exert anti‐inflammatory effects on the vascular cells. This system could facilitate testing of new drugs with human‐relevant metabolic profiles. These findings will have great impact on drug development and broad applications in studying diseases implicating vascular‐liver paracrine interaction.

## Introduction

Cardiovascular diseases and stroke, which are among the top causes of mortality, pose major health care burdens. Common risk factors such as high blood pressure and hypercholesterolemia underscore a predisposition to blood vessel dysfunction. Atherosclerosis, characterized by arterial hardening and fatty plaque build‐up in vessel walls, is one of the key contributors to vascular pathology. It is multifactorial, involving many cell types in a complex interplay of inflammation and oxidative stress [1]. Long‐term medication is often required for secondary prevention of adverse vascular events. Despite beneficial pleiotropic effects of cholesterol‐lowering statins on the vasculature, other side effects have been reported [2]. The concept of medical nutrition is on the rise to help modulate chronic diseases [3]. Dietary phospholipids from soybean, eggs, and fish have been explored as nutraceuticals with protective effects on atherosclerosis [4]. Herbal extracts are used as antiatherogenic agents by reducing the production, absorption, or oxidation of cholesterol [5, 6]. Unlike small‐molecule drugs, the complexity of constituents in nutraceuticals often present challenges in predicting their efficacy in preclinical screening. The liver is integral to biotransformation of complex compounds into new chemical species that may confer either therapeutic or toxic effects. Therefore, a human‐relevant vascular model that incorporates liver metabolism would be more effective in assessing bioactivity of nutraceutical ingredients.

Advances in human pluripotent stem cell (hPSC) differentiation offer an unparalleled ability to generate many cell types in adequate quantities for tissue engineering, regenerative medicine, and disease modeling in vitro [7, 8, 9]. Vascular cell‐based phenotypic assays have been demonstrated using endothelial and smooth‐muscle cells generated from hPSCs [10, 11, 12]. In response to inflammatory or biomechanical stimuli, hPSC‐derived endothelial cells are able to model athero‐susceptible phenotypes and display a breach of barrier integrity to allow leukocyte transmigration. Although incorporation of primary hepatocytes is most likely to replicate human liver function for drug testing in a metabolism‐enabled cellular model, their scarcity and the high cost of freshly isolated or cryopreserved human primary hepatocytes limit their extensive use [13]. We and others have created hPSC‐derived hepatocytes that express cytochrome P450 (CYP) drug‐metabolizing enzymes and show sensitivity to drugs with known hepatotoxicity [14, 15]. Interestingly, induced hepatocytes derived directly by lineage reprogramming of human fibroblasts have comparable CYP activities with primary hepatocytes [16]. Vascularized liver microtissue has been proposed, although its utility as a drug‐testing platform has not yet been shown [17]. Thus far, there has not been a model developed to recapitulate vasculature‐liver paracrine interaction for efficacy testing of complex compounds.

In this work, we developed a vascular‐liver model based on hPSC‐derived endothelial cells (hPSC‐ECs) and hPSC‐derived hepatocytes (hPSC‐HEPs). The hPSC‐ECs, generated via a mesodermal precursor population, demonstrated functional characteristics such as tube formation and inflammatory activation. HPSC‐HEPs were derived by using our previous protocol [18], and they expressed drug‐metabolizing enzymes. To test our hypothesis, we selected two nutraceuticals, quercetin and genistein, that are known to have potential vascular‐protective effects [19, 20]. We found that the parent compounds elicited minimal anti‐inflammatory effects on hPSC‐ECs. Conversely, when cocultured with hPSC‐HEPs, the inflammatory response of hPSC‐ECs was suppressed effectively, suggesting nutraceutical bioactivation by the metabolic activity of hepatocytes. Our data support the hypothesis that hPSC‐HEPs could process quercetin and genistein into primary and secondary metabolites, which exerted greater anti‐inflammatory effects compared with their parent compounds. Hence, this hPSC‐based endothelial‐hepatic platform could better predict the efficacy of nutraceuticals and identify beneficial properties of their constituent ingredients.

## Materials and Methods

### HPSC Culture and Maintenance

HPSCs were passaged by using a gentle cell dissociation agent (StemCell Technologies, Vancouver, BC, Canada, http://www.stemcell.com, catalog no. 07174) and seeded onto Matrigel‐coated plates in mTeSR1 medium (StemCell Technologies, catalog no. 05850). WA09 human embryonic stem cells (H9‐ESCs) and iPSCs derived from IMR90 fibroblasts (IMR90‐iPSCs) were purchased from WiCell (Madison, WI, http://www.wicell.org). iPSCs derived from BJ fibroblasts (BJ‐iPSCs) were kindly provided by a collaborator’s laboratory.

### Generation of Endothelial Cells From hPSCs

**Figure 1:**
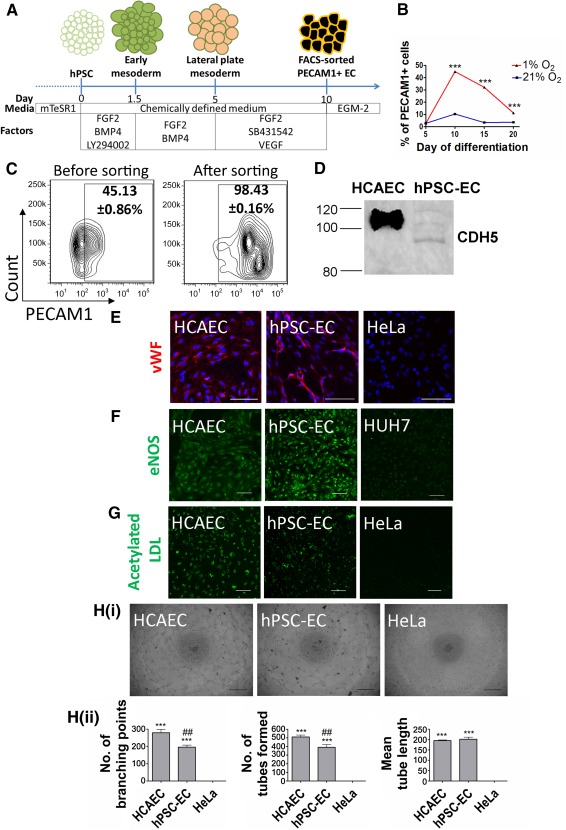
Generation and characterization of functional endothelial cells from human embryonic stem cells. **(A):** Timeline of endothelial induction from hPSC, delineating the differentiation media and factors used. HPSC‐ECs were derived via a lateral plate mesoderm population on day 5. By day 10, PECAM1‐expressing cells could be isolated and expanded further. **(B):** Flow‐cytometric analysis demonstrating that 1% O_2_ promoted higher percentages of PECAM1‐expressing cells than 21% O_2_, during endothelial induction from day 5 onward. ∗∗∗, *p* < .001 (compared with 21% O_2_; *n* = 3 independent biological replicates). **(C):** Flow cytometric contour plots on the purity of PECAM1‐expressing cells after FACS. **(D):** Western blot staining for CDH5 protein in cell lysates of hPSC‐ECs and HCAEC. **(E):** Immunostaining for endothelial marker vWF in hPSC‐ECs, HCAEC (positive control) and HeLa cells (negative controls). **(F):** Immunostaining for functional marker eNOS in hPSC‐ECs, HCAEC, and HUH7 cells (negative control). **(G):** Uptake of 3,3'‐dioctadecyloxacarbocyanine‐labeled acetylated‐LDL in hPSC‐ECs, HCAECs, and HeLa cells. Scale bars = 100 µm. **(Hi):** Phase contrast images of tube formation at 3 hour postseeding. Scale bars = 500 µm. **(Hii):** Quantification of tube forming capability of cells. ∗∗∗, *p* ≤ .001 relative to HeLa; ##, *p* ≤ .01 relative to HCAEC (*n* = 3 independent biological replicates). Abbreviations: BMP4, bone morphogenetic protein 4; EC, endothelial cell; eNOS, endothelial nitric oxide synthase; FACS, fluorescence‐activated cell sorting; FGF2, fibroblast growth factor 2; HCAEC, human coronary artery endothelial cell; hPSC, human pluripotent stem cell; hPSC‐EC, human pluripotent stem cell‐derived endothelial cell; HUH7, human hepatocellular carcinoma cells; LDL, low‐density lipoprotein; LY294002, phosphoinositide 3‐kinase inhibitor; PECAM1, platelet and endothelial cell adhesion molecule 1; VEGF, vascular endothelial growth factor; vWF, von Willebrand factor.

**Figure 2:**
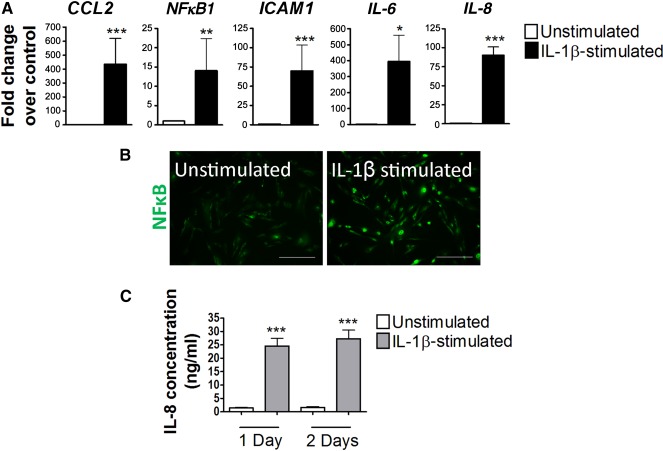
Inflammatory activation of H9‐embryonic stem cell‐derived endothelial cells by IL‐1β. **(A):** Upregulation of inflammatory genes in human pluripotent stem cell‐derived endothelial cells (hPSC‐ECs) stimulated by IL‐1β (20 ng/ml) for 6 hours. **(B):** Immunostaining of NFκB showed nuclear translocation in hPSC‐ECs after IL‐1β (20 ng/ml) for 1 hour. Scale bars = 100 µm. **(C):** Enzyme‐linked immunosorbent assay quantification of IL‐8 concentrations in conditioned media of IL‐1β‐stimulated hPSC‐ECs. ∗, *p* ≤ .05; ∗∗, *p* ≤ .01; ∗∗∗, *p* ≤ .001 (compared with their respective unstimulated controls; *n* = 3 independent biological replicates). Abbreviations: IL, interleukin; NFκB1, nuclear factor κB1.

**Figure 3:**
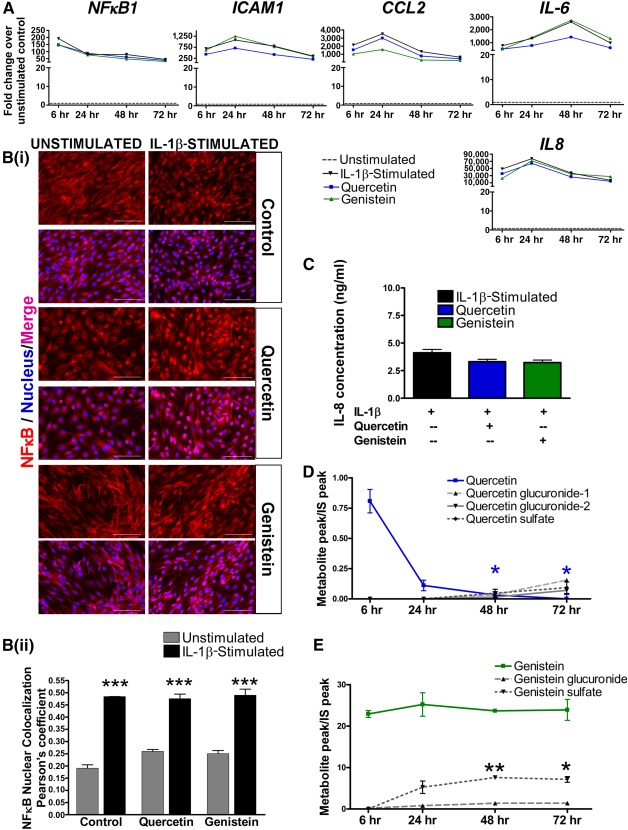
Quercetin and genistein are unable to suppress IL‐1β‐induced inflammation in H9‐embryonic stem cell‐derived endothelial cells (ECs). **(A):** Gene expression of inflammatory markers in IL‐1β‐stimulated human pluripotent stem cell‐derived endothelial cells (hPSC‐ECs) treated with quercetin and genistein over time. **(Bi):** Immunostaining for NFκB in unstimulated and IL‐1β‐stimulated hPSC‐ECs, treated with or without quercetin and genistein. Scale bars = 100 μm. **(Bii):** Quantification of nuclear colocalization levels of NFκB by Pearson’s coefficient. ∗∗∗, *p* ≤ .001 (compared with their respective unstimulated controls; *n* = 3 independent biological replicates). **(C):** Enzyme‐linked immunosorbent assay quantification of IL‐8 concentrations in conditioned media of IL‐1β‐stimulated hPSC‐ECs, treated with or without quercetin and genistein for 48 hours. **(D, E):** Liquid chromatography‐mass spectrometry analysis of metabolic profiles of parent nutraceuticals, quercetin (blue line) and genistein (green line) by hPSC‐ECs. Primary and secondary metabolites of nutraceuticals were indicated by black lines. ∗, *p* ≤ .05; ∗∗, *p* ≤ .01 (compared with their respective 6‐hour time points; *n* = 3 independent biological replicates). Abbreviations: IL, interleukin; IS, internal standard; NFκB, nuclear factor κB.

**Figure 4:**
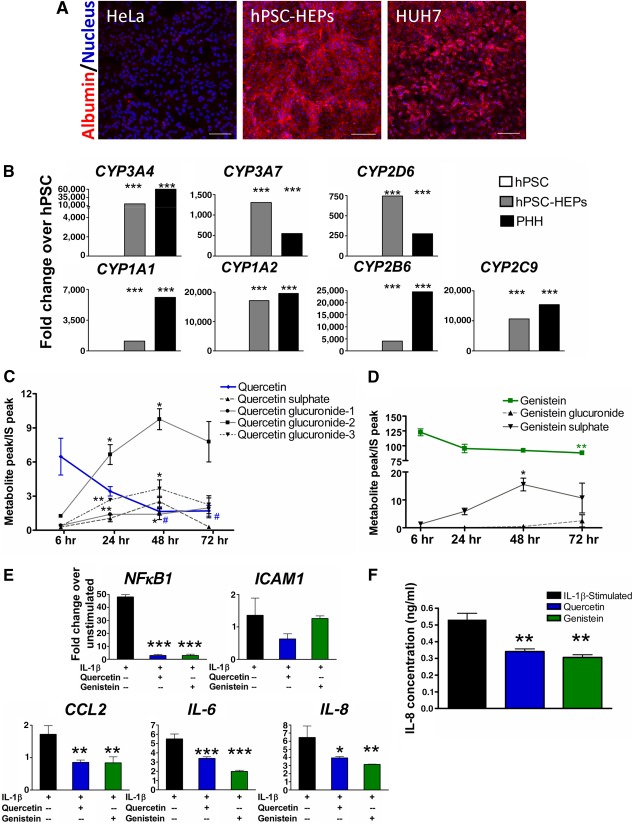
H9‐embryonic stem cell‐derived hepatocytes (HEPs) are able to effectively metabolize nutraceuticals. **(A):** Immunocytochemistry showing presence of albumin in hPSC‐HEPs and HUH7 cells (positive control), but not in HeLa cells (negative control). Scale bars = 200 μm. **(B):** Characterization of cytochrome P450 gene expression in hPSC‐HEPs, PHH (positive control), and hPSC (negative control). **(C, D):** Liquid chromatography‐mass spectrometry analysis of metabolic profiles of parent nutraceuticals, quercetin (blue line) and genistein (green line) by hPSC‐HEPs. The production of primary and secondary metabolites of nutraceuticals (indicated by black lines) increased with time. ∗, *p* ≤ .05; ∗∗, *p* ≤ .01 (compared with their respective 6‐hour time points; *n* = 3 independent biological replicates). **(E):** Gene expression levels of inflammatory markers in IL‐1β‐stimulated hPSC‐HEPs upon nutraceutical treatment for 48 hours. Quercetin (blue bar) or genistein treatments (green bar) decreased most of the inflammatory marker expression in stimulated hPSC‐HEPs. ∗, *p* ≤ .05; ∗∗, *p* ≤ .01; ∗∗∗, *p* ≤ .001 (compared with stimulated group without nutraceutical treatment (black bar); *n* = 3 independent biological replicates). **(F):** Enzyme‐linked immunosorbent assay quantification of IL‐8 concentrations in conditioned media of IL‐1β‐stimulated hPSC‐HEPs treated with or without quercetin and genistein for 48 hours. ∗∗, *p* ≤ .01 (compared with stimulated group without nutraceutical treatment; *n* = 3 independent biological replicates). Abbreviations: CYP3A4, cytochrome P450 3A4; hPSC, human pluripotent stem cell; hPSC‐HEPs, human pluripotent stem cell‐derived hepatocytes; HUH7, human hepatocellular carcinoma cells; IL, interleukin; IS, internal standard; NFκB1, nuclear factor κB1; PHH, primary human hepatocytes.

**Figure 5:**
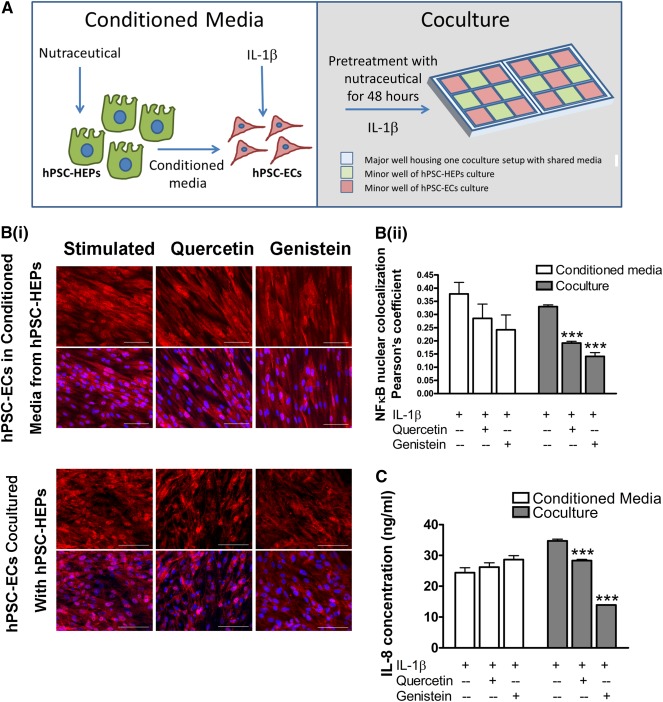
Coculture with H9‐embryonic stem cell‐derived hepatocytes (H9‐ESC‐HEPs) abrogates inflammatory activation in IL‐1β‐stimulated H9‐embryonic stem cell‐derived endothelial cells (H9‐ESC‐ECs). **(A):** Schematics showing two experimental setups to study endothelial‐hepatic paracrine interactions. **(Bi):** Immunostaining for NFκB in unstimulated and IL‐1β‐stimulated hPSC‐ECs, treated with or without quercetin and genistein. NFκB, red; nucleus, blue. Scale bars = 100 μm. **(Bii):** Quantification of NFκB nuclear translocation shows that quercetin or genistein significantly decreased levels of NFκB nuclear colocalization in coculture of hPSC‐ECs with hPSC‐HEPs. **(C):** IL‐8 protein levels were significantly reduced in endothelial‐hepatic coculture but not in conditioned media setup. Statistical differences were compared with their respective stimulated groups without nutraceutical treatment. ∗∗∗, *p* ≤ .001 (*n* = 3 independent biological replicates). Abbreviations: hPSC‐ECs, human pluripotent stem cell‐derived endothelial cells; hPSC‐HEPs, human pluripotent stem cell‐derived hepatocytes; IL, interleukin; NFκB, nuclear factor κB.

After the hPSC colonies attached, they were first induced to drive early mesoderm differentiation for 36 hours, by using a chemically defined medium, supplemented with human recombinant fibroblast growth factor 2 (FGF2) (20 ng/ml, R&D Systems, Minneapolis, MN, https://www.rndsystems.com, catalog no. 233‐FB), LY294002 (10 μM, Sigma‐Aldrich, St. Louis, MO, http://www.sigmaaldrich.com, catalog no. L9908), and human recombinant bone morphogenetic protein 4 (BMP4) (10 ng/ml, R&D Systems, catalog no. 314‐BP) [21]. Lateral plate mesoderm was further induced for another 3.5 days in medium supplemented with human recombinant FGF2 (20 ng/ml) and BMP4 (50 ng/ml), with medium change every 2 days. On day 5, the lateral plate mesoderm population was trypsinized by using TrypLE Express (Thermo Fisher Scientific Life Sciences, Oakwood Village, OH, https://www.thermofisher.com, catalog no. 12604) and plated on Matrigel‐coated plates in the same basal medium supplemented with FGF2 (4 ng/ml), SB431542 (10 μM, Sigma‐Aldrich, catalog no. S4317), and vascular endothelial growth factor (VEGF) (50 ng/ml, R&D Systems, catalog no. 293‐VE) with medium change every 2–3 days. On day 10, platelet and endothelial cell adhesion molecule 1 (PECAM1)‐expressing endothelial cells were sorted by using fluorescence‐activated cell sorting with PECAM1 antibody (BioLegend, San Diego, http://www.biolegend.com, catalog no.303110). The PECAM1+ cells were plated at a density of 5 × 10^4^ per cm^2^ onto collagen I‐coated plates in commercial medium EGM‐2 (Lonza, Walkersville, MD, http://www.lonza.com, catalog no. CC‐3162). hPSC‐ECs were passaged by using TrypLE Express when they reached more than 75% confluence. Fresh complete EGM‐2 medium was replaced every 2–3 days. All experiments were performed on cells up to passage 10. In this work, hPSC‐ECs derived from three different cell lines—H9‐ESCs (Figs. 1–5), IMR90‐iPSCs, and BJ‐iPSCs (supplemental online Figs. 2 and 3)—were functionally characterized and used in inflammatory activation assays. In EC marker and functional characterization, human coronary artery ECs (HCAECs) were used as positive control, whereas HeLa cells and human hepatocellular carcinoma cells (HUH7) were used as negative controls.

### Generation of Hepatocytes From hPSCs

Hepatocytes were generated from hPSCs by a growth factor‐based differentiation protocol described in our previous protocol [15]. After 20 days of differentiation, the cells were harvested by using a serial 2× TrypLE Express treatment and further dissociated into single cells by passing them through a 40‐μm cell strainer. These single cells were then seeded at 2.5 × 10^5^ cells per well in a collagen I (50 μg/ml, Bio Laboratories, Singapore, http://www.biolab.com.sg, catalog no. 354236)‐coated dishes. Attachment and recovery were promoted by seeding them in step IV differentiation medium with hepatocyte growth factor (R&D Systems, catalog no. 294‐HGN‐005), Follistatin (R&D Systems, catalog no. FS‐288), Oncostatin (R&D Systems, catalog no. 295‐OM‐010), and Y‐27632 (Rock Inhibitor) to prevent anoikis in the freshly harvested hPSC‐HEPs. The next day, medium was changed to Williams E medium (Sigma‐Aldrich, catalog no. W1878) without serum, and cells were serum‐starved overnight before nutraceutical treatments. Nutraceuticals quercetin (Sigma‐Aldrich, catalog no. Q4951) and genistein (Sigma‐Aldrich, catalog no. G6649) were administered at a single dose of 10 μM. Hepatocytes in this work were derived from H9‐ESCs. In hepatic characterization, primary human hepatocytes (PHHs) and HUH7 cells were used as positive controls, and HeLa cells were used as negative controls.

### HPSC‐HEP‐Conditioned Media Experiments on hPSC‐ECs

HPSC‐HEPs (1.25 × 10^5^ cells per cm^2^) were cultured for 48 hour in 1:1 William’s E medium + EGM‐2 without serum containing 10 μM quercetin [22, 23, 24, 25] or 10 μM genistein [22, 26, 27, 28, 29, 30, 31]. Conditioned medium from hPSC‐HEPs treated with quercetin or genistein was collected and added to hPSC‐ECs (that were serum‐starved overnight) along with 20 ng/ml interleukin‐1β (IL‐1β) for determination of nuclear factor κB (NFκB) nuclear colocalization (at 1 hour), gene expression profile of inflammatory markers (at 6 hours), and IL‐8 protein levels (1 day) upon inflammation.

### Endothelial‐Hepatocyte Coculture Experiments on Ibidi 2 × 9 Wells

H9‐ESCs were used to generate the ECs and hepatocytes for coculture experiments and assays with nutraceuticals. Both cell types were derived from an isogenic source of hPSCs to ensure a robust endothelial‐hepatic model without confounding phenotypic differences due to genetic variations. The Ibidi μ‐slide 2 × 9 wells (Ibidi, Martinsried, Germany, http://ibidi.com) were used for creating the coculture setup. Each minor well was first coated with collagen I, and then a total of 2.5 × 10^5^ hPSC‐ECs and hPSC‐HEPs were seeded in individual wells in a 1:1 ratio [32] within a major well to allow equivalent contribution of paracrine factors between the two cell types (Fig. 5A). After cell attachment, the medium was changed to 1:1 William’s E and EGM‐2 without serum, to be commonly shared by hPSC‐ECs and hPSC‐HEPs in a major well. Upon overnight serum starvation, the cocultures were preconditioned with 10 μM quercetin or genistein for 48 hours before IL‐1β stimulation.

### Real‐Time Quantitative Polymerase Chain Reaction

Total RNA was prepared by using an RNeasy mini kit (Qiagen, Hilden, Germany, http://www.qiagen.com, catalog no. 74104). Each RNA sample (250 ng) was reverse‐transcribed into cDNA by using the Maxima First Strand cDNA Synthesis kit (Thermo Fisher Scientific, catalog no. K1641). Quantitative polymerase chain reaction was performed on a StepOnePlus Real‐Time PCR system (Thermo Fisher) by using FAST SYBR green master mix (Thermo Fisher, catalog no. 4385616). Expression levels were normalized to the housekeeping gene glyceraldehyde 3‐phosphate dehydrogenase. Refer to supplemental online Table 1 for primer sequences.

### Flow Cytometry

Cells were trypsinized and stained by using 5 μl of anti‐human CD31 antibody (BioLegend, catalog no. 303110) diluted in 80 μl of phosphate‐buffered saline (PBS) with 20% fetal bovine serum (FBS) per 10^6^ cells, for 1 hour at 4°C, after which cold PBS was used to wash the cells. The cell pellet was collected by centrifugation at 200*g* for 3 minutes. The cell pellet was resuspended in 350 µl of PBS with 20% FBS for sorting. PECAM1+ cells were sorted by using a FACSAria IIu SORP cell sorter (BD Biosciences, San Jose, CA, http://www.bdbiosciences.com) and collected in PBS containing 20% FBS.

### Immunocytochemistry

Cells were fixed with 4% paraformaldehyde (Nacalai Tesque, Kyoto, Japan, http://www.nacalai.co.jp, catalog no. 09154‐14) and permeabilized by 0.5% Triton X‐100 (Acros Organics, Geel, Belgium, http://www.acros.com, catalog no. 215680010) in PBS with Ca^2+^ and Mg^2+^ at room temperature. Blocking was performed by using PBS with 10% serum overnight at 4°C. Cells were incubated with the respective primary antibody diluted in 0.1% serum containing PBS for 1 hour and secondary antibodies in 0.1% serum containing PBS for another hour. Washes were performed twice using 0.1% serum containing PBS. The 4′,6‐diamidino‐2‐phenylindole (1 μg/ml, Thermo Fisher, catalog no. D3571) was used to stain the cell nucleus for 10 minutes. Refer to supplemental online Table 2 for details for primary antibodies used.

### Tube Formation Assay

Matrigel‐coated plates for tube formation were prepared by adding 50 μl of Matrigel (10 mg/ml, BD Biosciences, catalog no. 356234) per well of a 96‐well plate and incubated at 37°C for 30 minutes. Cells were trypsinized and plated at a density of 9.3 × 10^4^ cells per cm^2^ in 150 μl of complete EGM‐2 per well. Images were taken hourly by using an inverted microscope (Olympus, Shinjuku, Tokyo, http://www.olympus‐global.com, CKX41) at ×5 magnification. Quantitative analysis of tube characteristics was performed by WimTube image processing online software (Wimasis Image Analysis, Córdoba, Spain, http://www.wimasis.com).

### Acetylated Low‐Density Lipoprotein Uptake Assay

The 3,3'‐dioctadecyloxacarbocyanine acetylated low‐density lipoprotein (Biomedical Technologies, Villalba, Spain, http://www.biomedical‐technologies.com, catalog no. BT‐925) was diluted in complete EGM‐2 at a concentration of 10 μg/ml before incubation with hPSC‐ECs at 37°C with 5% CO_2_ for 4 hours. The cells were visualized and imaged by using an Olympus Fluoview inverted confocal microscope at ×20 magnification.

### Enzyme‐Linked Immunosorbent Assay

Conditioned EGM‐2 medium was collected, and the concentration of human IL‐8 was determined by using the human IL‐8 enzyme‐linked immunosorbent assay (ELISA) kit (Thermo Fisher, catalog no. KHC0081), according to the manufacturer’s instructions.

### Western Blot

The cell lysates were collected by using radioimmunoprecipitation assay buffer (Thermo Fisher, catalog no. 89901) containing 1× proteinase inhibitor cocktail (Sigma‐Aldrich, catalog no. P8340). Protein quantification was performed by using the Quant‐iT protein assay kit (Thermo Fisher, catalog no. Q32210). A total of 80 μg of cell lysates was separated by NuPAGE 10% Bis‐Tris Gel (Thermo Fisher, catalog no. NP0303BOX) and transferred onto a nitrocellulose membrane. MagicMark XP Western protein standard (Thermo Fisher, catalog no. LC5602) was used to determine the molecular weight of protein bands. The WesternDot 625 goat anti‐rabbit Western blot kit (Thermo Fisher, catalog no. W10142) was used to visualize the protein bands. Blocking was performed at 4°C overnight by using 3% skimmed milk in 1× wash buffer provided by the kit and stained with CDH5 antibody in 3% skimmed milk solution for 1 hour at room temperature. The protein bands were visualized and imaged by using Bio‐Rad ChemiDoc MP Imaging System (Bio‐Rad, Hercules, CA, http://www.bio‐rad.com).

### Liquid Chromatography‐Mass Spectrometry

The metabolic potential of the hPSC‐HEPs and primary rat hepatocytes (freshly isolated according to our previously established protocol [33]) were tested by exposing them to nutraceuticals quercetin and genistein (10 μM) over different durations. Internal standard (IS) (Emodin, 10 ng/ml) was added to the conditioned medium. The solid‐phase extraction column (Phenomenex, Torrance, CA, https://www.phenomenex.com, Strata C18‐E, 55 μm, 70A) was conditioned by washing with 1 ml of methanol and then 2 ml of deionized water. Conditioned medium was added into the column, and 1.5 ml of 30% methanol was added to elute the impurity such as phenol red in the medium. 0.1% formic acid methanol was added to the column to elute all the metabolites and internal standard out to a 15‐ml tube. Liquid in the 15‐ml tube was dried under N_2_ in a sample concentrator with 30°C heater. After drying the sample, 100 µl of 0.1% formic acid methanol was added to the 15‐ml tube and vortex for 30 seconds and transferred to another 1.5‐ml tube. The samples were then centrifuged at 13,000 rpm for 10 minutes at 4°C, and 10 µl of the supernatant was injected into liquid chromatography‐mass spectrometry (LC‐MS). High‐performance liquid chromatography combined with electrospray ionization (ESI) ion trap time‐of‐flight (IT‐TOF) multistage mass spectrometry analyses were performed with a Shimadzu LC‐MS‐IT‐TOF instrument, which was composed of two LC‐20AD pumps, a SIL‐20AC autosampler, a CTO‐20A column oven, a CBM‐20A system controller, an ESI ion source, and an IT‐TOF mass spectrometer (Shimadzu, Kyoto, Japan, http://www.shimadzu.com).

### Statistical Analysis

Data were expressed as mean ± SD of at least three biological replicates of independent experiments. Statistical comparisons were conducted by Student’s unpaired *t* test with 95% confidence interval for two groups of samples or one‐way analysis of variance with Bonferroni’s post hoc test in multiple group comparisons. Analyses were carried out with GraphPad Prism 5 software (GraphPad Software Inc., La Jolla, CA, http://www.graphpad.com).

## Results

### Derivation of Functional Endothelial Cells From Human Pluripotent Stem Cells

Lateral plate mesoderm is a precursor tissue of vascular lineages. We adopted our established protocol of using fibroblast growth factor 2, bone morphogenetic protein 4, and phosphoinositide 3‐kinase inhibitor (LY294002) to induce lateral plate mesoderm for 5 days [21] (Fig. 1A). We then used a combination of factors to drive endothelial specification. Transforming growth factor β (TGF‐β) inhibition using small molecule SB431542 has been shown to enhance endothelial differentiation of hPSCs [34, 35], possibly by counteracting growth of mural cells, which could arise from a common cardiovascular progenitor. FGF2 and vascular endothelial growth factor are commonly known mitogens for promoting angiogenesis and endothelial development [36, 37]. Studies have also shown that hypoxic conditions increase the efficiency of endothelial differentiation [38, 39], because upregulation of hypoxia‐inducible factor triggers downstream targets that play an important role in early blood vessel development [40]. To induce endothelial differentiation, the day 5 mesodermal population was dissociated and plated down as single cells. We cultured these cells under 1% oxygen (O_2_) in a chemically defined medium containing SB431542, FGF2, and VEGF (Fig. 1A). Endothelial genes were significantly enhanced in 1% O_2_ as compared with 21% O_2_, peaking primarily at approximately day 10 (supplemental online Fig. 1A). Flow‐cytometric analysis further supported that endothelial specification was optimal at approximately day 10 in 1% O_2_, with more than 45% of the cells positive for PECAM1 (Fig. 1B; supplemental online Fig. 1B). This protocol generated a sufficient yield of PECAM1+ cells for cell sorting on day 10 of differentiation, giving rise to a purity of 98.43 ± 0.16% (Fig. 1C). This PECAM1+ population was then grown on collagen I coating and expanded by using a commercial endothelial growth media (EGM‐2). We hereafter refer to these cells as hPSC‐ECs.

Western blot demonstrated the presence of endothelial adherens junctions, CDH5, in hPSC‐ECs and the positive control, human coronary artery endothelial cells (Fig. 1D). Different glycosylated forms of CDH5 were found in hPSC‐ECs, close to the molecular weights of those in HCAEC. We postulated that there might be differences in glycosaminoglycan synthesis enzymes in hPSC‐ECs and HCAECs [41]. Our hPSC‐ECs formed spontaneous tube structures that stained for the mature endothelial marker, von Willebrand factor (vWF) (Fig. 1E). In addition, hPSC‐ECs possessed endothelial nitric oxide synthase (eNOS) (Fig. 1F) and were capable of taking up acetylated low‐density lipoprotein (LDL) (Fig. 1G), resembling HCAECs, but not the negative controls. We observed comparable tube‐forming capability between hPSC‐ECs and HCAECs (Fig. 1H). We reproduced this endothelial differentiation protocol on two other hPSC lines, namely, the BJ‐ and IMR90‐induced pluripotent stem cells. The BJ‐ and IMR90‐derived ECs also expressed endothelial proteins (supplemental online Fig. 2A, 2B), as well as demonstrated tube formation capability (supplemental online Fig. 2C). These functional hPSC‐ECs were subsequently used for assay development.

### HPSC‐Derived Endothelial Cells Respond to Inflammatory Stimulation

Inflammation is a hallmark of atherosclerosis [1]. To recapitulate atherosclerosis‐associated phenotypes in hPSC‐ECs, we used an inflammatory cytokine, interleukin‐1β, which is widely implicated in atherosclerosis. Upon stimulation with human recombinant IL‐1β, hPSC‐ECs responded with a significant upregulation of inflammatory genes (Fig. 2A). Nuclear translocation of nuclear factor κB, activating major proinflammatory mediators, has been observed in human atherosclerotic lesions [42]. Likewise, nuclear translocation of NFκB was evident in hPSC‐ECs after stimulation with IL‐1β (Fig. 2B). The production of interleukin 8 from conditioned media of IL‐1β‐stimulated hPSC‐ECs was significantly higher than that of the unstimulated cells (Fig. 2C). In addition to H9‐ECs, we also validated that BJ‐ECs and IMR90‐ECs could respond to IL‐1β by upregulation of inflammatory genes and increase of NFκB nuclear translocation, as well as elevated IL‐8 production (supplemental online Fig. 3). Hence, we were able to monitor hPSC‐EC inflammatory activation using a range of phenotypic readouts.

### Nutraceuticals Are Not Effective in Suppressing the Inflammatory Response of hPSC‐Derived Endothelial Cells

Next, we tested whether administration of nutraceuticals quercetin and genistein could suppress inflammatory responses in IL‐1β‐stimulated hPSC‐ECs. Quercetin, a naturally occurring flavonoid compound, is found commonly in food, such as tea, onions, berries, and apples. It exerts various beneficial effects through its anti‐inflammatory [19] and antioxidant [43] properties. Quercetin intake is also correlated with lower incidence of coronary heart disease and stroke [44]. Genistein, a potent phytoestrogen, is effective in mitigating endothelial dysfunction [20] and exerts an anti‐inflammatory effect by downregulating the NFκB pathway [22]. Plasma concentrations for genistein can range from 0.03 to 16.34 μM [30, 45], in line with the dosage commonly used for in vitro studies [22, 26, 27, 28, 29, 30, 31]. Quercetin has very low bioavailability in human plasma, where the concentrations range between 0.3 and 3.5 μM [46, 47, 48, 49]. Nonetheless, higher concentrations of quercetin are known to be safe and well tolerated [50, 51]. Previous studies in human hepatocytes [24, 25] and human C‐reactive protein mice [23] have used quercetin at a concentration of 10 μM. Therefore, we chose to treat the stimulated hPSC‐ECs with quercetin or genistein at a concentration of 10 µM for up to 72 hours. However, gene expression of inflammatory markers did not show substantial reduction from the IL‐1β‐stimulated levels at various time points (Fig. 3A). NFκB nuclear translocation levels remained elevated despite administration of quercetin and genistein (Fig. 3B). There was also no significant reduction of IL‐8 protein levels from the conditioned media of stimulated hPSC‐ECS after nutraceutical treatment for 48 hours (Fig. 3C).

We further investigated whether the hPSC‐ECs could metabolize the nutraceuticals. By using liquid chromatography‐mass spectrometry to analyze conditioned media from hPSC‐ECs, the level of quercetin (blue line) was found to decrease over time, whereas the levels of metabolites (black lines) from both nutraceuticals did not increase remarkably (Fig. 3D, 3E). Our data showed that first, the parent compounds may not be effective at eliciting anti‐inflammatory effects on endothelial cells. Second, limited capacity of hPSC‐ECs to break down the nutraceuticals into metabolites could have compromised the bioactivity of these compounds. Therefore, we explored whether hepatocytes derived from hPSCs were capable of metabolizing the nutraceuticals.

### HPSC‐Derived Hepatocytes Bioactivate Nutraceuticals Through Metabolism

Liver has been shown to metabolize quercetin into its bioactive metabolites, which in turn exert greater beneficial effects compared with their parent compound [24, 25]. We generated hepatocytes from hPSCs following our established protocols [15, 18]. The stepwise differentiation protocol recapitulates embryonic liver development because hPSCs progressively turn from primitive streak/mesoendoderm, definitive endoderm, and hepatoblasts to become hepatocytes (hereafter referred to as hPSC‐HEPs). Our hPSC‐HEPs stained positive for albumin, characteristic of functional hepatocytes (Fig. 4A). In accordance with our previous findings [15], we produced hPSC‐HEPs that expressed cytochrome P450 genes (Fig. 4B), some of which were comparable to the positive control PHH. CYP enzymatic activities are necessary for the metabolism of complex compounds.

Indeed, LC‐MS analysis demonstrated that the levels of both quercetin (blue line) and genistein (green line) gradually declined over time in the presence of hPSC‐HEPs (Fig. 4C, 4D). Correspondingly, the levels of metabolites (black lines) increased over time in the hPSC‐HEPs. Most of the metabolites peaked at 48 hours and dropped by 72 hours. Thus, hPSC‐HEPs were capable of converting quercetin and genistein into their metabolites, with 48 hours being the optimal duration based on the metabolic profiles of these nutraceuticals. When we compared the metabolic activity of our hPSC‐HEPs to freshly isolated primary rat hepatocytes [33], both demonstrated that quercetin (blue bars) and genistein (green bars) declined over time, giving rise to metabolites (gray patterned bars) (supplemental online Fig. 4). Because the primary rat hepatocytes could have retained some in vivo characteristics, their metabolic kinetics was apparently faster because substantial metabolites had emerged by 6 hours of treatment. We then investigated the effects of nutraceuticals on IL‐1β‐stimulated hPSC‐HEPs. We observed a significant reduction in the panel of inflammatory gene expression upon nutraceutical treatment (Fig. 4E). The production of IL‐8 protein from conditioned media of hPSC‐HEPs was also significantly suppressed after treatment with nutraceuticals for 48 hours (Fig. 4F). Hence, the ability of hPSC‐HEPs to process nutraceuticals into their bioactive metabolites could have resulted in their efficacy in abrogating inflammatory activation.

### Renewal of Nutraceutical Metabolites in the Presence of Hepatocytes Protects Endothelial Cells From Inflammatory Activation

To enable accurate assessment of complex compounds in vascular health, we examined two configurations of endothelial‐hepatic paracrine interaction. First, we allowed 48‐hour preincubation of each nutraceutical with hPSC‐HEPs for metabolism to take place (Fig. 5A). Subsequently, hPSC‐HEP‐conditioned media were collected and treated on hPSC‐ECs under IL‐1β stimulation. Alternatively, we cocultured hPSC‐ECs and hPSC‐HEPs on IBIDI μ‐slide 2 × 9 wells, where each cell type could be seeded separately into minor wells and shared a common medium by filling up the major wells (Fig. 5A). The coculture was pretreated with each nutraceutical for 48 hours, followed by IL‐1β stimulation. Our data showed that hPSC‐HEP‐conditioned media with either quercetin or genistein did not seem to inhibit NFκB nuclear translocation in IL‐1β‐stimulated hPSC‐ECs (Fig. 5B). In contrast, when cocultured with hPSC‐HEPs, stimulated hPSC‐ECs displayed a significant suppression of NFκB nuclear translocation. Furthermore, the secretion of IL‐8 in the coculture setting was remarkably decreased, but not in the conditioned media configuration (Fig. 5C). The metabolite profiles of quercetin (blue bar) and genistein (green bar) in each of the two configurations showed that there were detectable levels of various metabolites (gray bars) in the endothelial‐hepatic coculture (supplemental online Fig. 5B), but not in the conditioned media setting (supplemental online Fig. 5A). This supports that the metabolites in hPSC‐HEP‐conditioned media could be degraded to a certain extent when they were subsequently treated on hPSC‐ECs under IL‐1β stimulation. This might lead to insufficient anti‐inflammatory effects. Conversely, we also investigated whether hPSC‐ECs could, in turn, impact the metabolic function of hPSC‐HEPs in a coculture setting. It has been reported that endothelial cells could improve hepatic function [52, 53, 54, 55] and provide some levels of hepatoprotection from acetaminophen toxicity [56]. In our study, cocultured hPSC‐HEPs had comparable albumin levels with monocultured hPSC‐HEPs (supplemental online Fig. 6A). Notably, there was significant increase of CYP gene expressions in cocultured hPSC‐HEPs (supplemental online Fig. 6B), suggesting that the presence of hPSC‐ECs could promote metabolic activity in hPSC‐HEPs. Therefore, a coculture of hPSC‐ECs and hPSC‐HEPs could better recapitulate the in vivo vascular‐liver systemic interactome, with renewal of metabolites by liver metabolism.

## Discussion

We have developed an endothelial‐hepatic system to predict the efficacy of nutraceuticals in vascular protection. Insights from developmental studies guided our hPSC differentiation strategy. We established a protocol for efficient generation of functional endothelial cells from a lateral plate mesoderm precursor. Endothelial specification was induced by using FGF2, VEGF, and 1% O_2_, all of which play roles in blood vessel development and angiogenesis [57]. Furthermore, small‐molecule SB431542, a potent antagonist of activin receptor‐like kinase, could enhance the efficiency of endothelial differentiation by inhibiting TGF‐β signaling [35], which would otherwise promote mural cell specification from mesoderm. These hPSC‐ECs were responsive to IL‐1β‐stimulated inflammation, but treatment with either quercetin or genistein was not able to offset the inflammatory activation. This was because of limited metabolic activity of hPSC‐ECs to break down the nutraceuticals into bioactive metabolites. Hence, it led us to postulate that hepatocytes from hPSCs possess metabolic capacity to enhance bioavailability of metabolites from quercetin and genistein. We generated functional hepatocytes according to our previously established protocol [15]. The hPSC‐HEPs, with high expression of CYP enzymes, were able to effectively metabolize quercetin and genistein into primary and secondary metabolites. Similar metabolites were detected when we compared the nutraceutical treatment on our hPSC‐HEPs with primary rat hepatocytes, as well as those metabolites described in primary human hepatocytes [24, 25, 26, 27, 28, 58, 59]. We recognize that the primary rat hepatocytes required shorter time to metabolize the parent nutraceutical, because they were freshly isolated and hence could have retained most of their in vivo functionality. Depending on the structural complexities, it is also likely that different nutraceuticals would have distinct metabolic profiles. The hPSC‐HEPs may require different treatment durations to release optimal levels of metabolites. This also highlights the importance of dosage response in hPSC‐HEPs, where a range of physiologically relevant concentrations could be tested.

Incorporation of hepatocytes to endothelial culture underpins a key novelty of this work. Notably, hPSC‐HEP‐conditioned media containing nutraceutical metabolites were not effective in suppressing inflammation in hPSC‐ECs. There could be a decline in the potency of metabolites from hPSC‐HEP‐conditioned media due to degradation. Instead, when hPSC‐ECs were cocultured with hPSC‐HEPs in a shared medium, we noticed that there was a significant reduction in inflammation. Continuous replenishment of metabolites in coculture setup recapitulated the systemic setting of liver paracrine effects on the vasculatures. Conversely, endothelial cells are known to improve hepatic function by promoting cell viability, synthesis of albumin and urea, and efficiency of the drug transporter system [52, 53, 54, 55]. Our hPSC‐ECs could, in turn, increase the metabolizing CYP enzyme activity in hPSC‐HEPs. Another advantage of this endothelial‐hepatic crosstalk is to recapitulate human‐relevant response where certain drug metabolism dynamics may not be accurately reproduced in animals [60]. Our current system could still have limitations just as other in vitro cell‐based platforms. The need for evaluating chronic exposure (i.e., >3 weeks) to drugs will involve further optimization to ensure viability and sustainable functionality of cells in long‐term culture. For high‐throughput drug‐testing efforts, scalability of hPSC‐derived cells may require improved conditions, such as supportive extracellular matrices or automation in bioreactors [61, 62, 63, 64], for robustness of production.

Multicellular coculture models [55, 65, 66] are gaining momentum for various applications. A recent study described the use of human umbilical vein endothelial cells to stabilize hepatoblastoma C3A cells and modulate drug‐induced hepatotoxicity [56]. Endothelial cells from hPSCs were used to vascularize liver constructs [52]. These are part of the advances to model liver vasculatures, using multiple stromal cell types alongside hepatocytes to develop liver lobule and sinusoid‐like structures [52, 53, 54, 55, 56, 57, 65, 67, 68, 69, 70], for hepatotoxicity screening and tissue‐engineering applications. We are the first to report a human stem cell‐derived endothelial‐hepatic platform for efficacy testing of complex compounds. Nonetheless, we could capitalize on our endothelial‐hepatic model for further development. Cells‐on‐chip in microfluidic‐based systems could provide the benefits of different flow dynamics, minimizing reagents used, and optical suitability for high‐content imaging of cells. Initial studies showed that endothelial cells [71, 72] and hepatocytes [17, 73] have improved functionality in perfusion cultures. Phenotypic assays could also be developed to capture different pathological readouts for efficacy testing, as well as toxicology assessment. A spectrum of assay endpoints for vascular injury and atherosclerosis may include endothelial dysfunction, oxidative stress, apoptosis, matrix remodeling, etc. Multiplexing of phenotypic readouts in multicellular models [17, 74] would add great value to the applications of coculture systems. In addition, our endothelial‐hepatic platform could be used for disease modeling involving paracrine crosstalk between liver and vasculature. Because the liver is integral to normal or dysfunctional lipid homeostasis, this interplay could influence vascular function [75]. Moreover, it is likely that lipid‐modifying nutraceuticals may involve liver metabolism to exert their actions on vascular tissue.

## Conclusion

Our hPSC‐based endothelial‐hepatic model represents a physiologically relevant cellular system for nutraceutical screening, enabled by the paracrine crosstalk between the two cell types. Such a coculture system will help dissect the modes of actions of complex compounds without the need to purify into their constituent ingredients. Furthermore, this human‐relevant platform could enable mechanistic interrogation of candidate compounds with liver‐ and vascular‐targeting therapeutic effects or toxicity prediction.

## Author Contributions

B.C.N.: conception and design, collection and/or assembly of data, data analysis and interpretation, manuscript writing, final approval of manuscript; Y.T.G.: collection and/or assembly of data, data analysis and interpretation, manuscript writing; H.L. and H.Y.: collection and/or assembly of data (for LC‐MS), data analysis and interpretation, final approval of manuscript; S.S.: conception of mesoderm differentiation, final approval of manuscript; C.C.: conception and design, financial support, data analysis and interpretation, manuscript writing, final approval of manuscript.

## Disclosure of Potential Conflicts of Interest

The authors indicated no potential conflicts of interest.

## Supporting information

Supporting Information
