# Supplementary material for: Human Stem Cell‐Derived Endothelial‐Hepatic Platform for Efficacy Testing of Vascular‐Protective Metabolites from Nutraceuticals
Source: Stem Cells Transl Med. 2016 Oct 7;6(3):851–63. doi: 10.5966/sctm.2016-0129 (PMC5442778; doi:10.5966/sctm.2016-0129)
Supplement: Supplementary file 1 — Supporting Information [file SCT3-6-0851-s001.pdf]

Supplementary Information

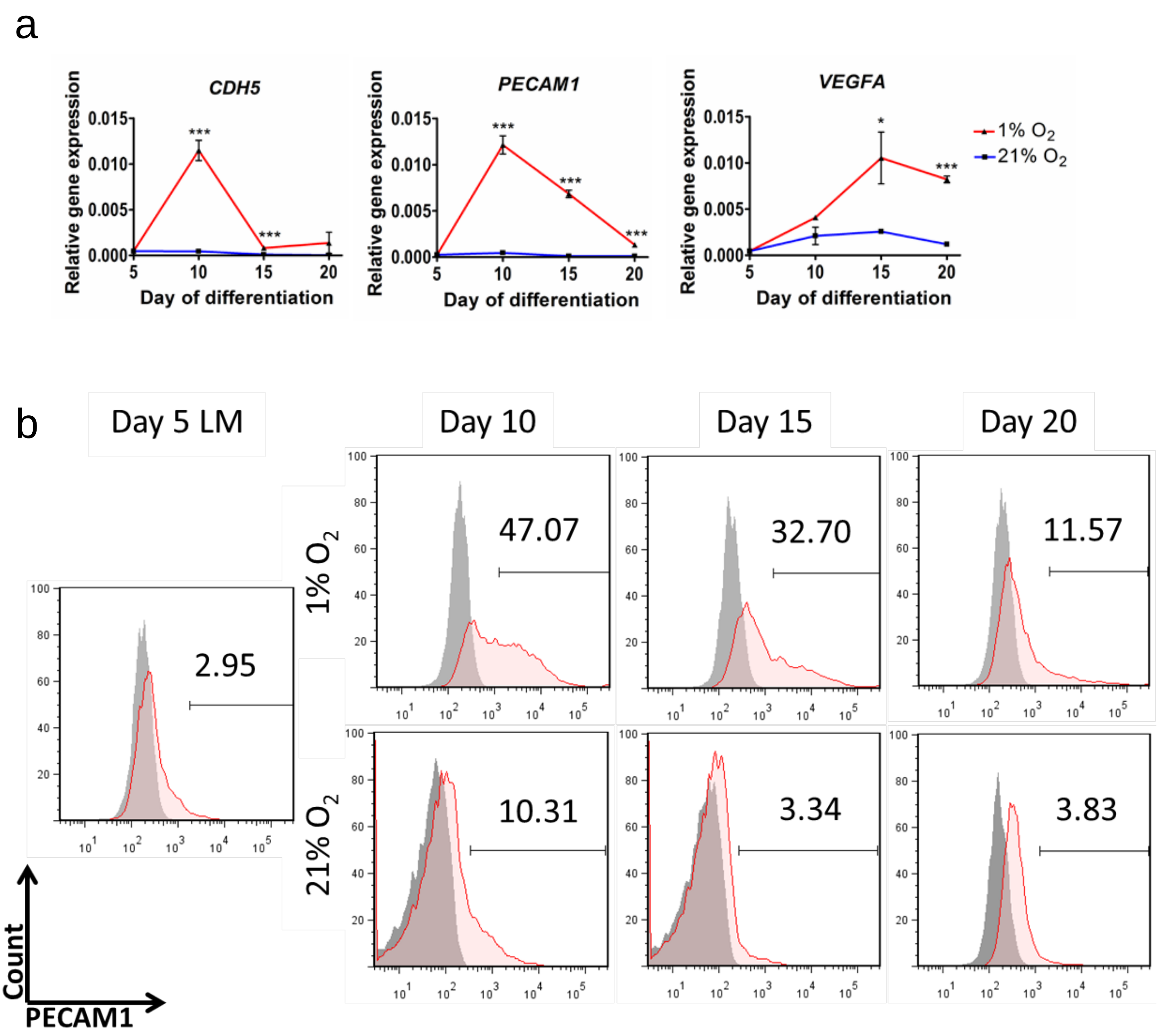

Supplementary Figure S1. Efficiency of endothelial differentiation in H9-ESCs. (**a**) Expression of endothelial genes over time under 1% or 21% O_2 culture. Data is represented as fold change over unstimulated control ± SEM. **p*-value <0.05 ****p*-value < 0.001. (**b**) Representative histogram plots of PECAM1-expressing cells during endothelial differentiation (day 10, 15, 20), under 1% O_2 vs 21% O_2 condition.

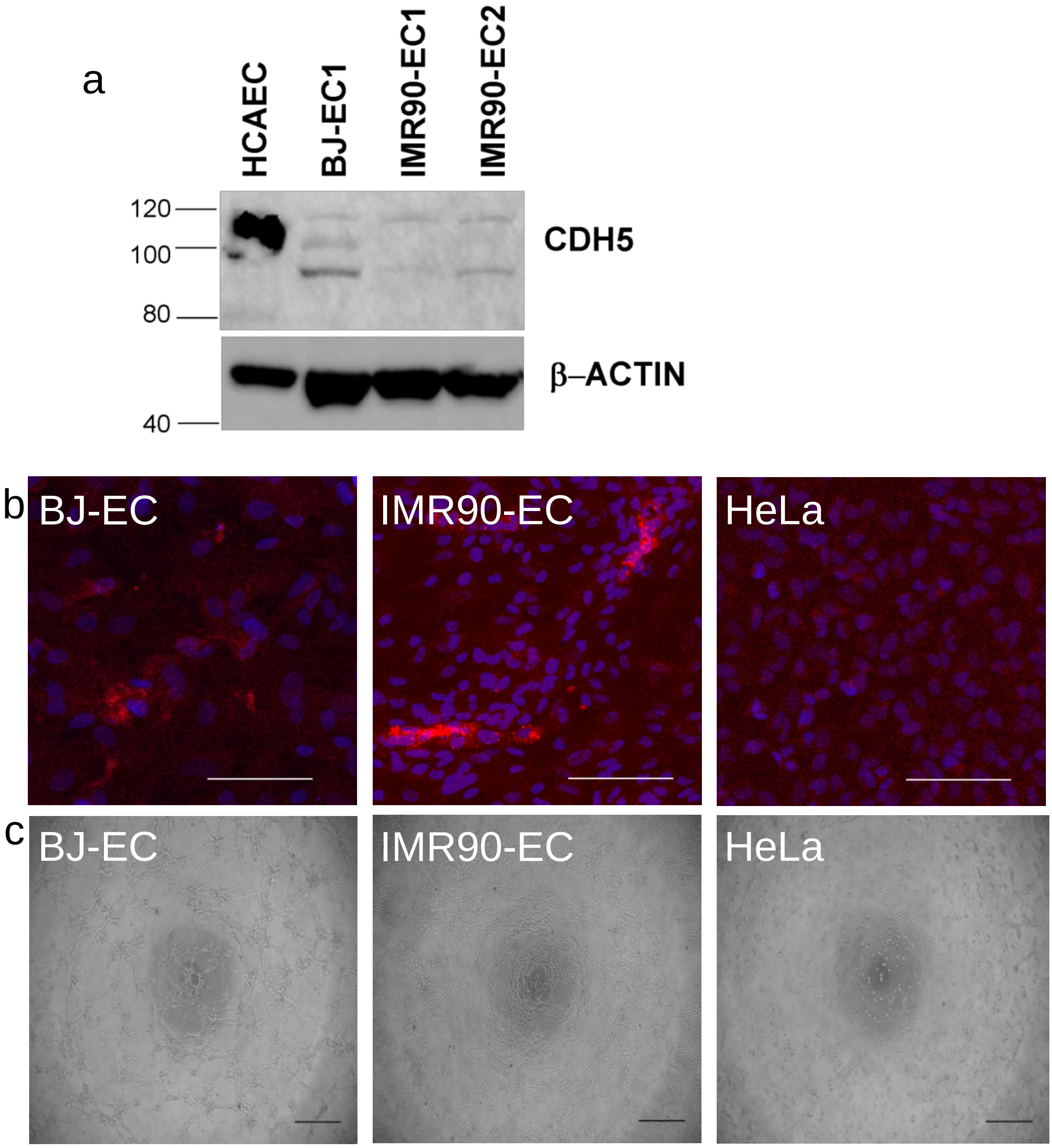

Supplementary Figure S2. Functional characterization of endothelial cells from BJ and IMR90 induced pluripotent stem cell lines. (**a**) Western blot of CDH5 protein in HCAEC, BJ-EC, IMR90-EC (duplicates). Glycosylated forms of CDH5 were detected. (**b**) Immunostaining of vWF in BJ-EC, IMR90-EC and HeLa cells (negative control). Scale bars, 100 µm. (**c**) Tube formation assay of BJ-EC and IMR90-EC seeded on matrigel plugs. Scale bars, 500 µm.

ScholarOne Support: (434) 964-4100

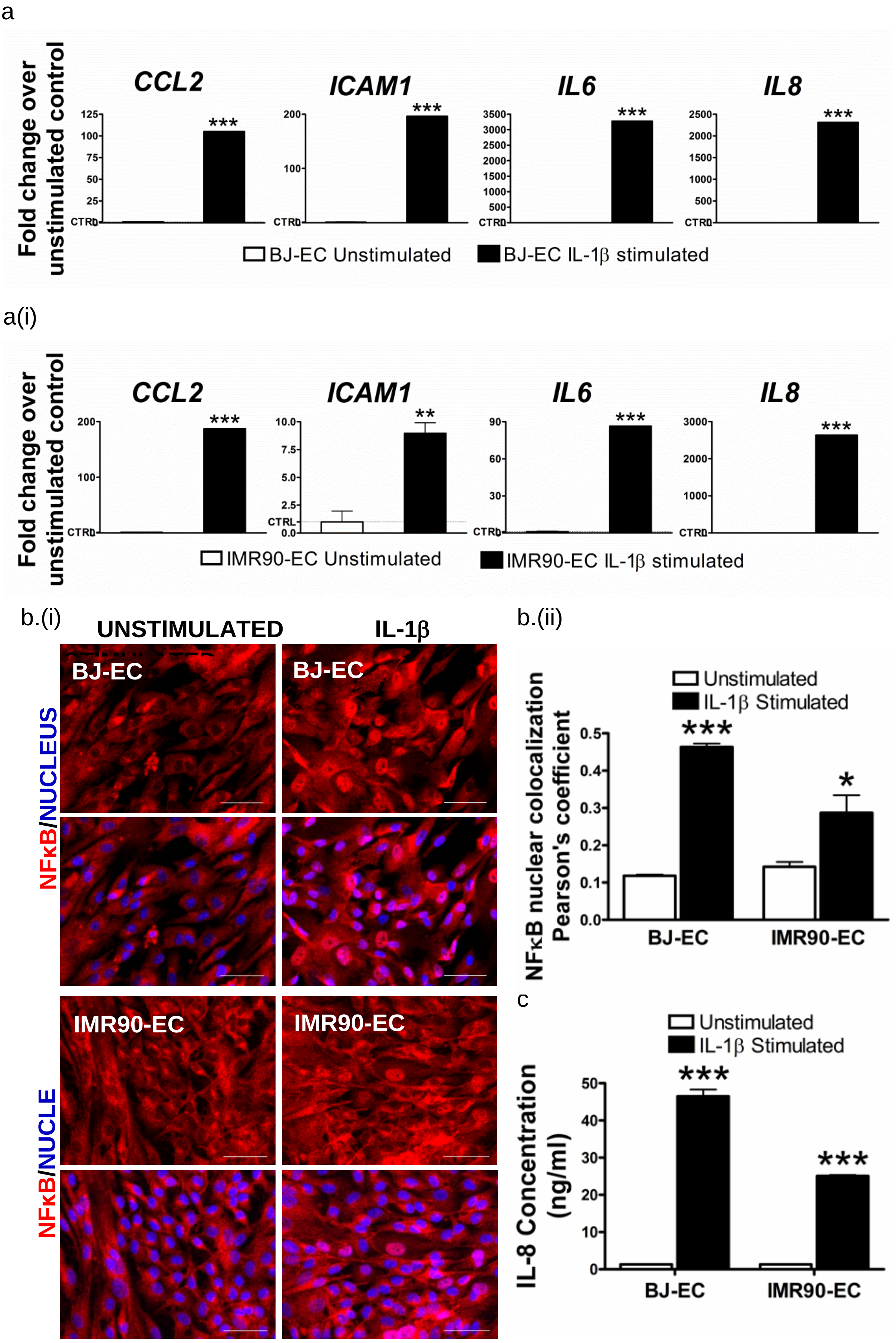
a
CCL2
ICAM1
IL6
IL8
Fold change over unstimulated control
BJ-EC Unstimulated
BJ-EC IL-1β stimulated
a(i)
IMR90-EC Unstimulated
IMR90-EC IL-1β stimulated
b.(i)
UNSTIMULATED
IL-1β
BJ-EC
IMR90-EC
NFκB/NUCLEUS
b.(ii)
Unstimulated
IL-1β Stimulated
NFκB nuclear colocalization Pearson's coefficient
BJ-EC
IMR90-EC
c
IL-8 Concentration (ng/ml)

Supplementary Figure S3: Inflammatory activation in BJ-ECs and IMR90-ECs. (a) Increased expression in inflammatory marker genes upon IL-1β stimulation in BJ-ECs and IMR90- ECs. Data is represented as fold change over unstimulated control ± SD. ***p*-value <0.01 ****p*-value < 0.001. **(b.i)** Immunofluorescent images of NFκB nuclear translocation upon IL-1β stimulation in BJ- ECs (top four panels) and IMR90- ECs (bottom four panels). Scale bar = 50 μm. **(b.ii.)** Quantification of immunofluorescent images shows significant increase in NFκB nuclear colocalization. **p*-value < 0.05, ****p*-value < 0.001. **(c)** Increased IL-8 protein levels in the IL-1β-stimulated BJ- ECs and IMR90- ECs. ****p*-value < 0.001.

ScholarOne Support: (434) 964-4100

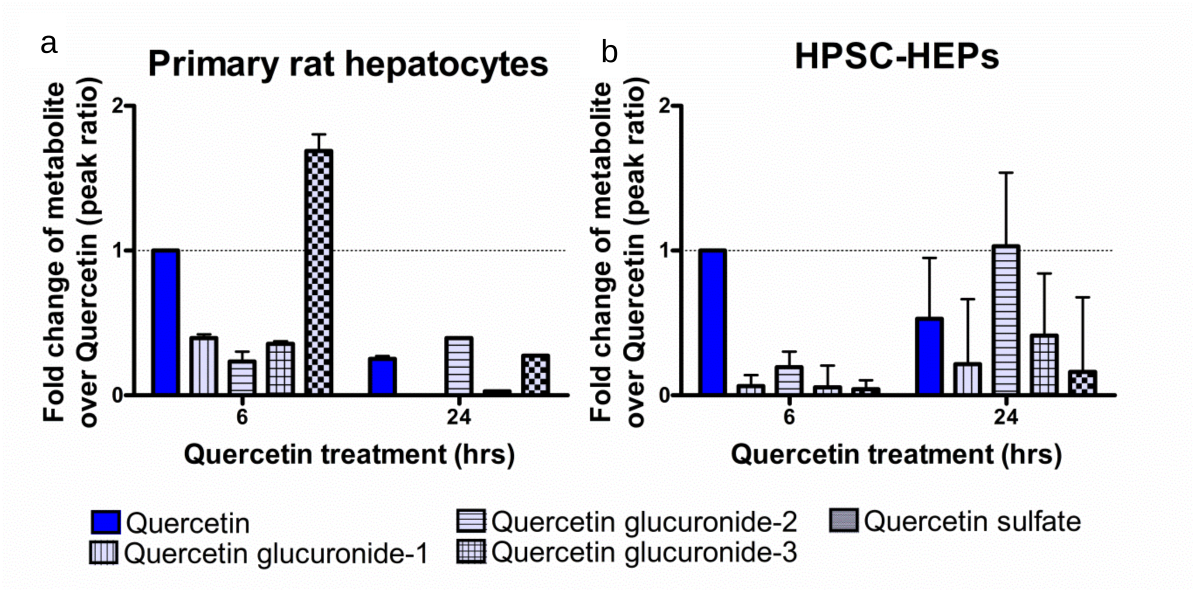

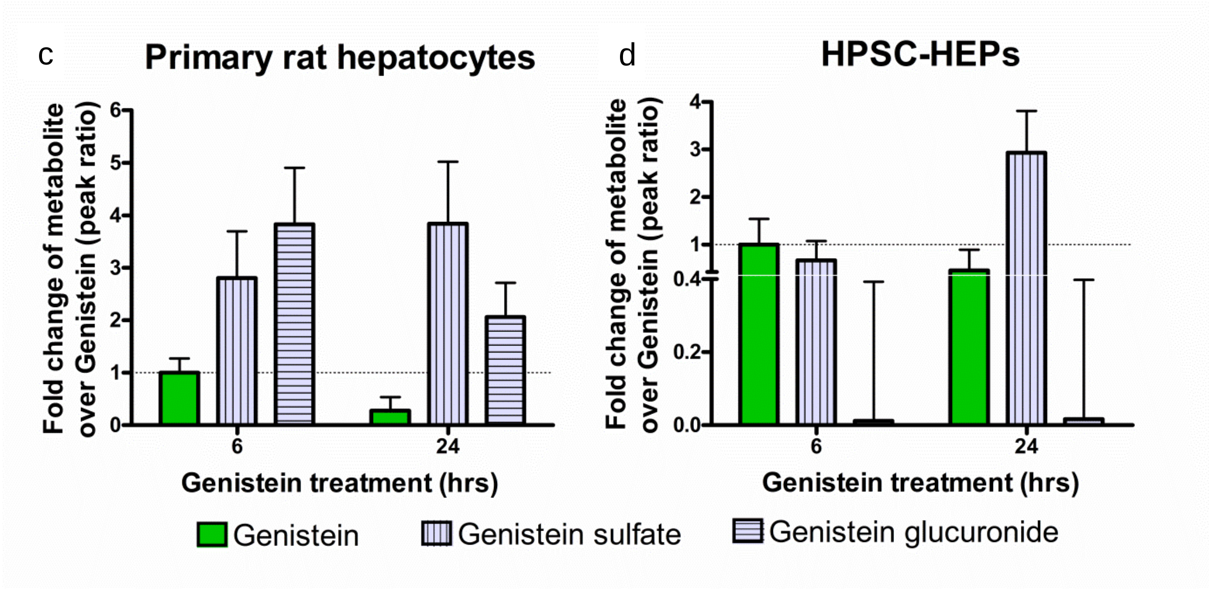

Supplementary Figure S4: Turnover of metabolites from quercetin (a,b) and genistein (c,d) in H9-ESC-HEPs and primary rat hepatocytes. Data is represented as fold change in the metabolite levels over parent metabolite at 6hrs, fold change in peak ratio (metabolite peak/IS peak) ± SD, n=3.

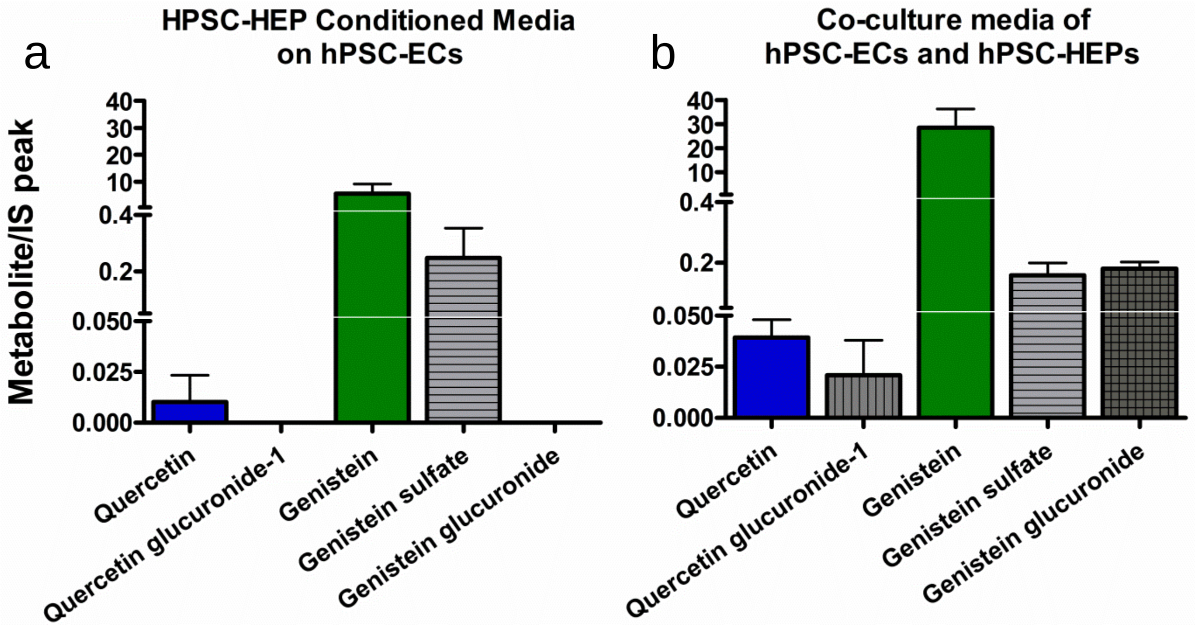

Supplementary Figure S5. LCMS analysis of quercetin and genistein metabolites in conditioned media and co-cultures. Metabolite profile of quercetin and genistein in (**a**) hPSC-HEP conditioned media treated on hPSC-ECs and (**b**) co-cultured media as measured by LCMS.

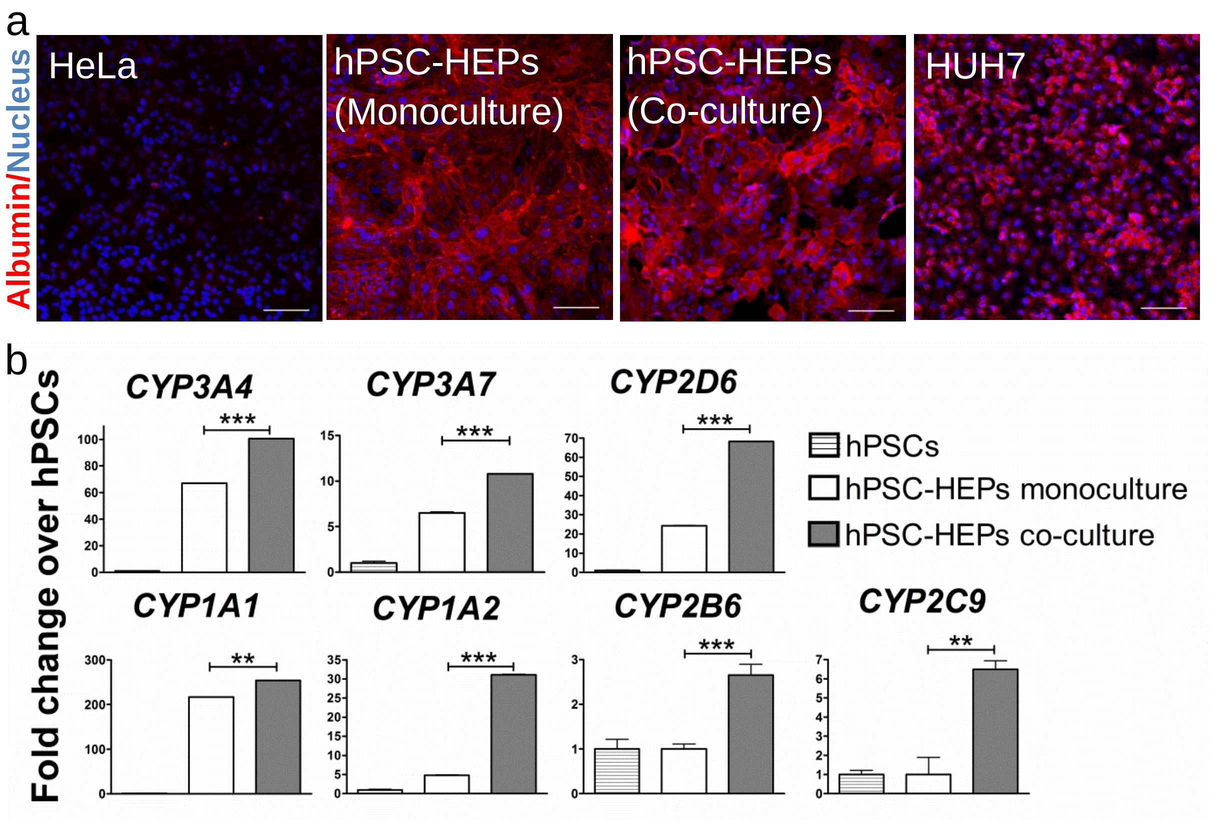

Supplementary Figure S6: Functional characterization of H9-ESC-HEPs co-cultured with H9-ESC-ECs. (a) Albumin immunofluorescence in hPSC-HEPs in monoculture and co-culture shows similar levels of expression in both the culture configurations. Scale bar = 100 μm. **(b)** The hPSC-HEPs co-cultured with hPSC-ECs express higher levels of CYP genes than the hPSC-HEPs in monoculture supporting the beneficial effects of endothelial cells on hPSC-HEPs metabolic potential. n=3. Data is represented as fold change over undifferentiated hPSCs ± SD. ***p*-value < 0.01, ****p*-value < 0.001.

Supplementary Tables

Supplementary Table 1 Primer sequences for qPCR

Genes	Sequences
CD31	CAGGCGCCGGGAGAAGTGAC
	CGTCCAGTCCGGCAGGCTCT
CDH5	TGGCCAGCTGGTCCTGCAGAT
	TGCCCGTGCGACTTGGCATC
TIE2	GCAGTGCAATGAAGCATGCCACC
	GGTAGCGGCCAGCCAGAAGC
CD34	CACAGGAGAAAGGCTGGGCGA
	TGGCCGTTTCTGGAGGTGGC
VWF	TAGCCCGCCTCCGCCAGAAT
	CCTGCAGGCGCAGGTGAAGT
VEGFa	GCACATAGGAGAGATGAGCTTC
	CCACAGGGACGGGATTTCTTG
NFKB1	ACTACCTGGTGCCTCTAGTGA
	TTTGACCTGAGGGTAAGACTTCT
MCP1	AAGCTCGCACTCTCGCCTCCA
	GCATTGATTGCATCTGGCTGAGCG
IL6	AAATTCGGTACATCCTCGACGG
	GGAAGGTTCAGGTTGTTTTCTGC
IL8	TTGGCAGCCTTCCTGATTTCTGCAG
	ACAACCCTCTGCACCCAGTTTTC
VCAM1	ATGGTCGCGATCTTCGGAGCC
	AACGGACTTGGCCCCCTCTGT
ICAM1	ACCGGAAGGTGTATGAACTGA
	TGGTTGGCTATCTTCTTGCAC
bFGF	AGCGACCCTCACATCAAGCTACA
	CTGCCCAGTTCGTTTCAGTGCCA
CYP3A4	AAGTCGCCTCGAAGATACACA
	AAGGAGAGAACACTGCTCGTG
CYP3A7	TGCTTTGTCCTTCCGTAAGGG
	CAGCATAGGCTGTTGACAGTC
CYP2B6	GCACTCCTCACAGGACTCTTG
	CCCAGGTGTACCGTGAAGAC
CYP1A1	ACATGCTGACCCTGGGAAAG
	GGTGTGGAGCCAATTCGGAT
CYP1A2	ATGCTCAGCCTCGTGAAGAAC
	GTTAGGCAGGTAGCGAAGGAT
CYP2C9	GCCTGAAACCCATAGTGGTG
	GGGGCTGCTCAAAATCTTGATG

ScholarOne Support: (434) 964-4100

Supplementary Table 2. Antibodies list

**Proteins**	**Applications**	**Dilution factors**	**Source**
NFκB	IMMUNO	1:200	ABCAM (AB32536)
VWF	IMMUNO	1:100	ABCAM (AB9378)
CDH5 (VE-cadherin)	WESTERN	1:1000	ABCAM (AB33168)
B-ACTIN	WESTERN	1:1000	SIGMA (SAB2100037)
eNOS	IMMUNO	1:100	ABCAM (AB5589)
Albumin	IMMUNO	1:100	R&D (MAB1455-SP)

ScholarOne Support: (434) 964-4100
